# Development and Evaluation of a Novel Upper-Limb Rehabilitation Device Integrating Piano Playing for Enhanced Motor Recovery

**DOI:** 10.3390/biomimetics10040200

**Published:** 2025-03-25

**Authors:** Xin Zhao, Ying Zhang, Yi Zhang, Peng Zhang, Jinxu Yu, Shuai Yuan

**Affiliations:** 1 School of Arts and Design, Yanshan University, Haigang District, Qinhuangdao 066000, China; cindy@ysu.edu.cn; 2 Arts Department of Qinhuangdao Vocational and Technical College, Beidaihe District, Qinhuangdao 066100, China; zhangying@qvc.edu.cn; 3 Organization and Publicity Office of the CPC Qinhuangdao Vocational and Technical College Committee, Beidaihe District, Qinhuangdao 066100, China; zhangyi@qvc.edu.cn; 4 Department of Design, Kyungpook National University, Daegu 41566, Republic of Korea; 2021327350@knu.ac.kr; 5 College of Mechanical and Automotive Engineering/Hangzhou Bay Automotive Engineering, Ningbo University of Technology, Ningbo 315211, China; jinxuyu@nbut.edu.cn

**Keywords:** upper-limb rehabilitation device, task-oriented occupational therapy, music-interactive rehabilitation

## Abstract

This study developed and evaluated a novel upper-limb rehabilitation device that integrates piano playing into task-oriented occupational therapy, addressing the limitations of traditional continuous passive motion (CPM) training in patient engagement and functional recovery. The system features a bi-axial sliding platform for precise 61-key positioning and a ten-link, four-loop robotic hand for key striking. A hierarchical control framework incorporates MIDI-based task mapping, finger optimization using an improved Hungarian algorithm, and impedance–admittance hybrid control for adaptive force–position modulation. An 8-week randomized controlled trial demonstrated that the experimental group significantly outperformed the control group, with a 74.7% increase in Fugl–Meyer scores (50.5 ± 2.5), a 14.6-point improvement in the box and block test (BBT), a 20.2-s reduction in nine-hole peg test (NHPT) time, and a 72.6% increase in rehabilitation motivation scale (RMS) scores (55.4 ± 3.8). The results indicate that combining piano playing with robotic rehabilitation enhances neuroplasticity and engagement, significantly improving motor function, daily activity performance, and rehabilitation adherence. This mechanical-control synergy introduces a new paradigm for music-interactive rehabilitation, with potential applications in home-based remote therapy and multimodal treatment integration.

## 1. Introduction

Stroke and peripheral nerve injuries are the primary pathological factors leading to upper-limb motor dysfunction. Patients typically exhibit symptoms such as weakened hand muscle strength, restricted joint range of motion, declined fine motor function, and impaired multi-joint coordination [1]. Upper-limb dysfunction severely affects patients’ daily living abilities and overall quality of life [2]. In rehabilitation therapy, effectively restoring upper-limb function, particularly the fine motor function of the hand, remains a significant focus and challenge in rehabilitation medicine and engineering.

Current upper-limb rehabilitation training methods primarily rely on physical and occupational therapy. However, traditional rehabilitation approaches have several limitations. Conventional rehabilitation equipment lacks engagement and is associated with poor patient compliance. Devices such as resistance bands, elastic balls, and basic mechanical rehabilitation apparatuses have simplistic designs and repetitive motion patterns, failing to sustain patient interest [3,4,5]. The repetitive nature of rehabilitation training often leads to patient fatigue, thereby reducing treatment adherence and training efficacy [6]. Traditional rehabilitation devices predominantly focus on single-joint training and lack the capability to facilitate multi-joint coordinated movements. Many rehabilitation devices target the functional recovery of isolated joints or specific regions, such as finger flexion or wrist extension [7]. However, restoring upper-limb motor function necessitates the coordinated movement of multiple joints, including the hand, wrist, elbow, and shoulder [8]. The absence of multi-joint coordination training in rehabilitation devices fails to meet the comprehensive recovery needs of patients and hinders the reconstruction of overall motor abilities [9]. Additionally, traditional rehabilitation devices often fail to sufficiently encourage patients’ initiative during rehabilitation training. These devices predominantly emphasize passive training, resulting in limited patient engagement and active participation [10]. Although passive training provides certain benefits in the early stages of rehabilitation, its long-term efficacy is limited and does not effectively harness patients’ intrinsic motivation. Furthermore, conventional rehabilitation training methods generally lack the ability to dynamically adjust training intensity, rhythm, and mode according to the patient’s rehabilitation progress.

On the other hand, the application of music therapy in neurological rehabilitation has been extensively studied and validated [11,12,13]. Music can promote neural function recovery and reconstruction by stimulating brain neuroplasticity [14]. The role of music therapy in motor function rehabilitation is primarily reflected in the following aspects: I. Neuroplasticity activation: music provides a unique neural stimulus, activating both motor and sensory areas of the brain through rhythm and melody [15]. The rhythmic structure of music can synchronize with the patient’s movement patterns, thereby optimizing motor coordination [16]. II. Enhanced patient engagement: music has an emotional influence that can improve a patient’s mood, alleviate psychological stress, and subsequently enhance participation in rehabilitation training and adherence to therapy [17]. III. Multisensory integration: music-based training involves auditory, visual, tactile, and motor perception pathways simultaneously, strengthening rehabilitation outcomes [18]. Specifically, piano playing, as a complex hand-coordination task, stimulates the auditory, visual, and motor systems simultaneously, thereby enhancing neuroplasticity. Additionally, piano playing offers a high level of engagement and immersion, which significantly boosts patient motivation and active participation [19].

However, while music therapy has been widely applied in lower limb gait training, such as music rhythm-based gait rehabilitation robots [20], its research in the field of upper-limb rehabilitation remains relatively limited, particularly in integrating musical elements with fine motor training. The development of rehabilitation devices that incorporate piano playing for upper-limb recovery is still in its early stages. Existing devices are primarily restricted to simple keyboard operations and lack comprehensive training mechanisms that engage shoulder, elbow, and finger movements in a coordinated manner [21].

Based on the aforementioned background, this study proposes a novel upper-limb occupational therapy device that integrates piano playing with upper-limb rehabilitation. The device features two linear sliding tracks positioned in front of an electronic keyboard, where a sliding platform supports the patient’s hand movement. This design facilitates coordinated motion training involving the hand, wrist, elbow, and shoulder, providing comprehensive upper-limb rehabilitation support. Additionally, a hand rehabilitation robot is incorporated to assist patients in executing precise key-pressing movements, enabling targeted finger motor training. This design not only enhances patient engagement and training experience but also optimizes upper-limb functionality through multi-joint coordinated movements.

## 2. Materials and Methods

### 2.1. Mechanical Structure Design

**Figure 1:**
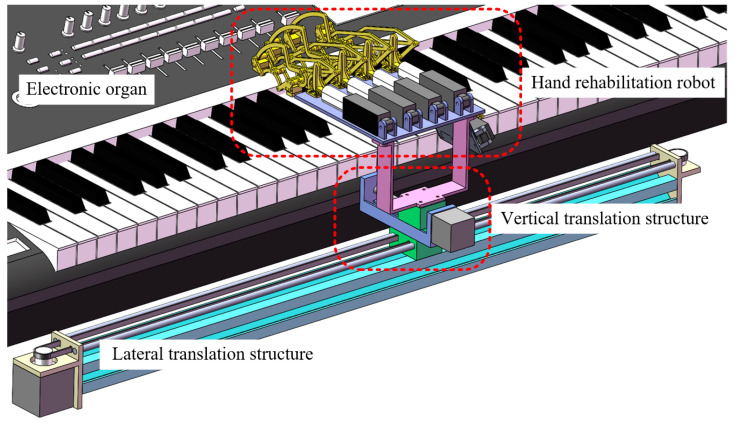
The specific structure of the novel upper-limb rehabilitation device.

The mechanical structure design of this study’s rehabilitation device aims to achieve multi-degree-of-freedom coordinated motion for upper-limb occupational therapy. By integrating piano playing with upper-limb rehabilitation, the device provides an efficient and engaging rehabilitation solution. The core design consists of two main components: the translational mechanism and the hand rehabilitation robot structure. The translational mechanism adopts a combined horizontal and vertical translation design, accommodating the spatial distribution differences between black and white piano keys. This allows for omnidirectional positioning of the patient’s hand within the electronic keyboard plane. Meanwhile, the hand rehabilitation robot structure assists patients in executing precise key-pressing movements. The specific structure of the equipment is shown in Figure 1. The following sections will elaborate on the design principles, technical details, and the rehabilitation applications of each component.

#### 2.1.1. The Horizontal Translation Mechanism

**Figure 2:**
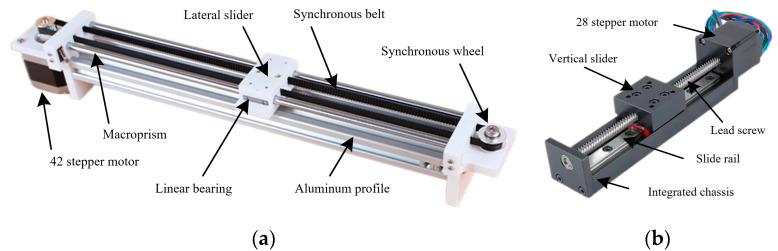
Structure of the translation mechanism: (**a**) horizontal translation; (**b**) vertical translation.

The horizontal translation mechanism provides rigid support for the main framework of the rehabilitation device, enabling the patient’s hand to move along the piano key arrangement direction (X-axis) while covering the entire keyboard range. It also bears the combined motion load of both the vertical translation mechanism and the hand rehabilitation robot, as shown in Figure 2a. The main framework of the horizontal translation mechanism is constructed using 6061-T6 aluminum alloy profiles with a cross-sectional dimension of 40 × 40 mm, ensuring a high bending stiffness of 2.1 × 10^4^ N·m^2^, as verified via ANSYS 2020R1static structural simulation. The horizontal translation mechanism consists of two independent linear motion units corresponding to the left and right hands of the patient. Each unit includes two cold-drawn steel guide rods (diameter 6 mm), surface-treated with hard chrome plating to achieve a roughness of Ra ≤ 0.4 μm. The guide rod length spans the full width of a 61-key electronic keyboard. The horizontal sliding platform is 3D-printed and embedded with two linear bearings (model LM6UU) that work in conjunction with the guide rods. The bearing preload is adjustable, ensuring a friction coefficient of ≤0.003. The drive system employs a NEMA 17 stepper motor with a holding torque of 0.45 N·m, coupled with a GT2 timing belt transmission with a belt pitch of 2 mm and a width of 5 mm. The closed-loop control system guarantees a repeatable positioning accuracy of ±0.1 mm.

#### 2.1.2. The Vertical Translation Mechanism

The vertical translation mechanism is nested within the horizontal sliding platform, with its primary function being to drive the hand along the depth direction of the piano keys (Y-axis) to accommodate the spatial position differences between black and white keys, as shown in Figure 2b. This mechanism employs a lead screw–guide rod hybrid transmission design, ensuring both high-motion precision and rapid response. The vertical drive system utilizes a lead screw with a 12 mm lead (precision grade C7) and a 6 mm diameter, which is directly coupled to a stepper motor. The effective stroke is 50 mm, covering the range of black key positions. The lead screw nut is rigidly connected to the vertical sliding platform, with preloading applied to eliminate backlash. A NEMA 11 stepper motor is selected as the drive motor, paired with a planetary gear reducer (reduction ratio 5:1), providing an output torque of 0.12 N·m. A magnetic encoder (resolution 0.01 mm) is integrated for real-time position feedback, and a PID control algorithm ensures a positioning accuracy of ±0.05 mm. The vertical sliding platform is fixed to the horizontal sliding platform via bolts, forming an XY-plane motion pair. The total weight of the vertical translation mechanism is controlled within 400 g to minimize excessive inertial load on the horizontal translation drive system.

#### 2.1.3. The Hand Rehabilitation Robot

**Figure 3:**
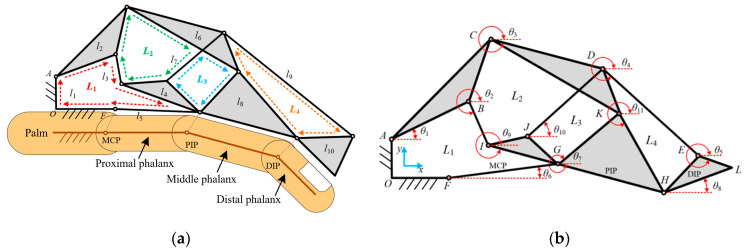
Transmission mechanism and kinematic analysis model of the hand rehabilitation robot: (**a**) transmission mechanism of the hand rehabilitation robot; (**b**) Lagrangian coordinate system of the transmission mechanism.

The hand rehabilitation robot is mounted on the vertical sliding platform and functions to drive the patient’s fingers in executing key-pressing movements through a biomimetic linkage mechanism. This robot adopts a modular design, allowing independent adjustment of training parameters for each finger. Each finger is controlled by an independent linear actuator with a 30 mm stroke, 20 N thrust, and an integrated magnetic encoder with a resolution of 0.05 mm. Each linear actuator used in the hand rehabilitation robot has a maximum speed of 24 mm/s, which is suitable for controlled and gradual finger movement during rehabilitation training. The speed was selected based on clinical studies on finger rehabilitation, ensuring that the movement is neither too fast to cause discomfort nor too slow to hinder efficient therapy. The control system allows real-time speed adjustment according to the patient’s progress and tolerance. The transmission mechanism is designed as a four-loop, ten-link mechanism, where the three finger segments of the human hand are treated as three deterministic links, referred to as finger segment links. Together with the input link position, a total of four loops are arranged as follows: I. Three finger actuation loops, each containing a finger segment link, which drives the corresponding finger segment to execute the desired motion; II. One drive input loop responsible for transmitting the actuator’s input force into the entire transmission system. The final four-loop, with the ten-link mechanism, consists of the following: I. One frame fixed to the outer casing of the hand rehabilitation robot. II. Three finger segment links corresponding to the three segments of the finger. III. Four compound links that optimize force transmission and structural integrity. This design not only enables multi-degree-of-freedom motion control but also significantly reduces the overall footprint of the transmission system by optimizing the spatial arrangement of the linkages. The resulting structure is more compact, lightweight, and streamlined, aligning with the design requirements for simplicity, efficiency, and minimalistic form factor. The structure of the hand rehabilitation robot is shown in Figure 3a.

First, the geometric method is employed to solve the minimal unit of each loop [22], followed by a position analysis of each loop. By leveraging the kinematic interdependencies among the loops, independent joint variables are correlated, leading to a complete kinematic solution of the mechanism. The proposed simplified linkage configuration can be decomposed into four independent loops: five-bar loop *L*_1_, five-bar loop *L*_2_, four-bar loop *L*_3_, and four-bar loop *L*_4_. For planar multi-loop kinematic analysis, a Lagrangian coordinate system is established for the transmission structure, as shown in Figure 3b. Then, the geometric method is applied to analyze the kinematics of each minimal loop unit. Subsequently, the kinematic relationships between loops are used to establish a comprehensive kinematic model of the mechanism.

**Figure 4:**
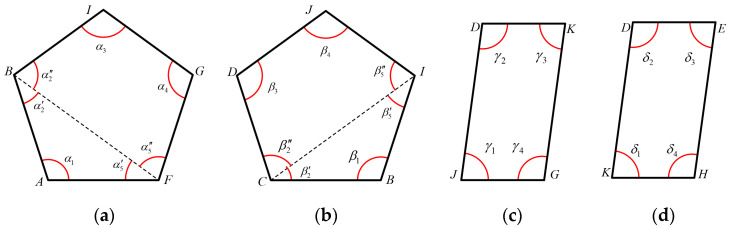
Four loops of the transmission mechanism for the hand rehabilitation robot: (**a**) Five-bar loop *L*_1_, (**b**) Five-bar loop *L*_2_, (**c**) Four-bar loop *L*_3_, and (**d**) Four-bar loop *L*_4_.

As shown in Figure 4a, the five-bar loop is divided into ∆ABF and four-bar loop BIGF by connecting point *B* and point *F*. Geometric methods can be used to obtain:

(1) $$l_{BF}=\sqrt{l_{AB}^{2}+l_{AF}^{2}-2l_{AB}l_{AF}\cos\alpha_{1}}$$

Substituting Equation (1) into the following equation can solve $\alpha_{5}^{\prime }$:

(2) $$\alpha_{5}^{\prime }=\arccos\left(\frac{l_{BF}^{2}+l_{AF}^{2}-l_{AB}^{2}}{2l_{BF}l_{AF}}\right)$$

From the sum of the interior angles of a triangle, we can obtain $\alpha_{2}^{\prime }$:

(3) $$\alpha_{2}^{\prime }=\pi -\alpha_{1}-\alpha_{5}^{\prime }$$

By using the vector method to analyze the four-bar loop BIGF, we can obtain the following:

(4) $${\vec{l}}_{IG}={\vec{l}}_{BF}+{\vec{l}}_{FG}-{\vec{l}}_{BI}$$

Simplifying it yields as follows:

(5) $$A_{1}\cos\alpha_{5}^{\prime \prime }+B_{1}\sin\alpha_{5}^{\prime \prime }+C_{1}=0$$

The algebraic variables $A_{1}$, $B_{1}$, and $C_{1}$ can be represented as follows:

(6) $$\left\{\begin{array}{l} A_{1}=2l_{BF}\left(l_{IG}\cos\alpha_{2}^{\prime \prime }-l_{BI}\right)\\ B_{1}=-2l_{IG}l_{BF}\sin\alpha_{2}^{\prime \prime }\\ C_{1}=l_{BI}^{2}+l_{IG}^{2}+l_{BF}^{2}-l_{GF}^{2}-2l_{BI}l_{IG}\cos\alpha_{2}^{\prime \prime } \end{array}\right.$$

Now, $\alpha_{5}^{\prime \prime }$ can be solved as follows:

(7) $$\alpha_{5}^{\prime \prime }=\arccos\left(\frac{-A_{1}C_{1}+B_{1}\sqrt{A_{1}^{2}+B_{1}^{2}-C_{1}^{2}}}{A_{1}^{2}+B_{1}^{2}}\right)$$

From the trigonometric formula, we can obtain the following:

(8) $$\alpha_{3}=\arccos\left(\frac{l_{IG}-l_{BI}\cos\alpha_{2}^{\prime \prime }+l_{BF}\cos(\alpha_{2}^{\prime \prime }+\alpha_{5}^{\prime \prime })}{l_{GF}}\right)$$

(9) $$\alpha_{4}=\arccos\left(\frac{l_{BF}-l_{BI}\cos\alpha_{3}+l_{IG}\cos(\alpha_{2}^{\prime \prime }+\alpha_{5}^{\prime \prime })}{l_{GF}}\right)$$

Based on the obtained interior angles and the sum of interior angles in a pentagon, the following can be concluded:

(10) $$\left\{\begin{array}{l} \alpha_{5}=\alpha_{5}^{\prime }+\alpha_{5}^{\prime \prime }\\ \alpha_{2}=\alpha_{2}^{\prime }+\alpha_{2}^{\prime \prime }\\ \alpha_{1}+\alpha_{2}+\alpha_{3}+\alpha_{4}+\alpha_{5}=3\pi \end{array}\right.$$

By combining Equations (6)–(9), the internal angle $\alpha_{2}^{\prime \prime }$ can be obtained. Then, by reverse substituting Equations (7)–(9), the values of internal angles $\alpha_{5}^{\prime \prime }$, $\alpha_{3}$ and $\alpha_{4}$ can be obtained. From this point on, all internal angles of the five-bar loop can be determined. The five-bar loop *L*_2_, as shown in Figure 4b, can also obtain kinematic closed-form solutions for each position according to the above process.

For the four-bar loop JDKG, as shown in Figure 4c, the vector method can be used to obtain the following:

(11) $${\vec{l}}_{DK}={\vec{l}}_{JG}+{\vec{l}}_{GK}-{\vec{l}}_{JD}$$

Simplifying it yields the following:

(12) $$A_{2}\cos\gamma_{4}+B_{2}\sin\gamma_{4}+C_{2}=0$$

The algebraic variables $A_{2}$, $B_{2}$ and $C_{2}$ can be represented as follows:

(13) $$\left\{\begin{array}{l} A_{2}=2l_{JG}(l_{DK}\cos\gamma_{1}-l_{JD})\\ B_{2}=-2l_{DK}l_{JG}\sin\gamma_{1}\\ C_{2}=l_{JD}^{2}+l_{DK}^{2}+l_{JG}^{2}-l_{KG}^{2}-2l_{JD}l_{DK}\cos\gamma_{1} \end{array}\right.$$

Now, $\gamma_{4}$ can be solved as follows:

(14) $$\gamma_{4}=\arccos\left(\frac{-A_{2}C_{2}+B_{2}\sqrt{A_{2}^{2}+B_{2}^{2}-C_{2}^{2}}}{A_{2}^{2}+B_{2}^{2}}\right)$$

From the trigonometric formula, we can obtain $\gamma_{3}$ as follows:

(15) $$\left\{\begin{array}{l} \gamma_{2}=\arccos\left(\frac{l_{DK}-l_{JD}\cos\gamma_{1}+l_{JG}\cos(\gamma_{1}+\gamma_{4})}{l_{KG}}\right)\\ \gamma_{3}=\arccos\left(\frac{l_{JG}-l_{JD}\cos\gamma_{4}+l_{DK}\cos(\gamma_{1}+\gamma_{4})}{l_{KG}}\right) \end{array}\right.$$

From then on, all interior angles of the four-bar loop *L*_3_ are determined. The four-bar loop *L*_4_, as shown in Figure 4d, can also obtain kinematic closed-form solutions for each position according to the above process.

To overcome the common issue in traditional linear actuators, where the actuator rod tilts upward during extension, leading to excessive spatial occupation and unstable operation [23]. We propose a parallel pushing structure. This design integrates a slider mechanism to ensure that the linear actuators maintain stable and parallel motion during operation, meeting the design requirements for precision and compactness.

**Figure 5:**
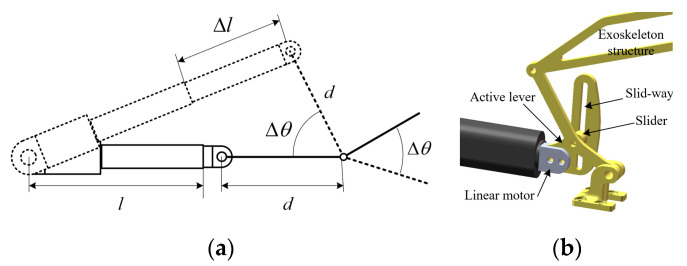
Analysis and solutions for the lifting problem of the push rod motor: (**a**) Analysis of the motor lifting problem; (**b**) Three-dimensional design drawing of the slider structure.

In conventional push rod actuators, when fully extended, the actuator rod often tilts upward due to its structural characteristics, increasing the overall space requirement, as shown in Figure 5a. To address this issue, we introduce a guided sliding structure, as shown in Figure 5b: I. A sliding groove is fixed at the head of the actuator. II. A bearing-integrated sliding block is attached at the connection point of the actuator rod. III. The sliding block moves within the groove, ensuring smooth and linear displacement, effectively eliminating the rod tilting issue. This parallel actuation design significantly enhances the stability, precision, and space efficiency of the system, making it well-suited for rehabilitation applications requiring precise and repeatable finger motion.

**Figure 6:**
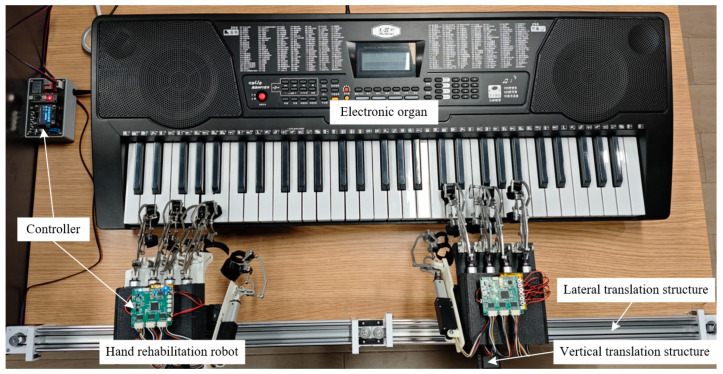
The specific structure of the newly designed upper-limb rehabilitation occupational therapy device integrated with piano playing.

The newly designed upper-limb rehabilitation occupational therapy device integrated with piano playing is shown in Figure 6.

### 2.2. Control Strategy Design

**Figure 7:**
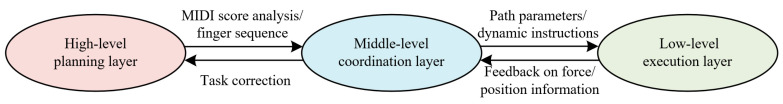
Hierarchical control flow chart.

To meet the control requirements for integrating music playing with hand rehabilitation occupational therapy, this study proposes a hierarchical control strategy that emphasizes task-oriented execution and dynamic adaptability. By decomposing the complex control problem into multiple logical layers, the proposed strategy achieves end-to-end precise control, seamlessly linking music analysis with mechanical execution. The overall control process is illustrated in Figure 7.

The hierarchical control framework consists of three interconnected layers: the high-level planning layer, the mid-level coordination layer, and the low-level execution layer. The high-level planning layer is responsible for music score analysis, note-to-mechanical action mapping, and task allocation, ensuring that musical information is accurately converted into mechanical execution commands. The mid-level coordination layer focuses on path planning, finger movement optimization, and dynamic parameter adjustment, refining movement trajectories and adapting fingering strategies in real time. Finally, the low-level execution layer implements motor actuation, force–position hybrid control, and real-time feedback processing, ensuring precise key-pressing actions while maintaining adaptability based on patient responses.

#### 2.2.1. High-Level Planning Layer: Music-to-Mechanical Action Mapping

In the high-level planning layer, the system first performs note parsing and musical feature extraction. Music-driven rehabilitation training requires the transformation of musical symbols into executable commands for the mechanical system. In this study, the Music21 library in Python 3.13.2 is utilized to parse MIDI files and extract key musical parameters, including pitch, duration, and intensity. Each note in a MIDI file corresponds to a pitch number (ranging from 0 to 127) [24], while the note duration (in seconds) is calculated based on the beats per minute (BPM) and note type as follows:

(16) $$T_{note}=\frac{60}{\mathrm{BPM}}\times D$$

BPM is the beats per minute of the music. *D* is the note duration factor that corresponds to different note types (whole note: *D* = 4; half note: *D* = 2; quarter note: *D* = 1; eighth note: *D* = 0.5; sixteenth note: *D* = 0.25; thirty-second note: *D* = 0.125) [25]. Formula (16) ensures that note duration is accurately converted from MIDI time format into real-world timing for mechanical execution.

MIDI files encode key-pressing intensity using a velocity value $V_{\mathrm{midi}}$, which ranges from 0 to 127. The key-pressing force $F_{\mathrm{key}}$ in Newtons is linearly mapped from this velocity value:

(17) $$F_{\mathrm{key}}=F_{\min}+\left(\frac{V_{\mathrm{midi}}}{127}\right)\times (F_{\max}-F_{\min})$$

$F_{\min}$ is the minimum pressing force (set to 1 N in this study), $F_{\max}$ is the maximum pressing force (set to 5 N in this study), and $V_{\mathrm{midi}}$ is the MIDI velocity (ranging from 0 to 127). Formula (17) ensures that a higher MIDI velocity value results in a greater key-pressing force, maintaining a natural and expressive playing experience while satisfying rehabilitation force requirements.

The physical parameters of the 61-key electronic keyboard used in this study are as follows:

- White keys: Width 23.5 mm (C1 to C6, 36 white keys in total).
- Black keys: Width 13 mm, positioned above the gaps between white keys.
- The horizontal position of the *n*th white key is calculated as:

(18) $$x_{k}^{\mathrm{white}}=23.5\times (n-1)\;(n=1,2,\ldots ,36)$$

- Black keys are located between adjacent white keys, with a lateral offset of +9.25 mm from the left white key and a fixed vertical offset of 12 mm.

A finger assignment algorithm is required to efficiently determine which finger should press a given key during piano playing. In this study, finger indexing is defined as follows: thumb (1), index finger (2), middle finger (3), ring finger (4), and little finger (5). To achieve optimal rehabilitation training, an enhanced Hungarian algorithm is introduced for finger optimization, formulating the assignment as a bipartite graph matching problem where left-side nodes represent notes to be played and right-side nodes correspond to available fingers. The cost function for finger *i* pressing note *j* is given by the following:

(19) $$c_{ij}=\alpha \cdot \|p_{i}-q_{j}\|+\beta \cdot \Delta t_{ij}+\gamma \cdot F_{\mathrm{norm}}$$

$\left\|p_{i}-q_{j}\right\|$ is the Euclidean distance from the current position of the finger $p_{i}$ to the target key $q_{j}$. $\Delta t_{ij}$ is the time required for the current state of the finger to switch to tapping $q_{j}$, $F_{\mathrm{norm}}$ is the normalization strength ($F_{\mathrm{norm}}=F_{\mathrm{key}}/5$), and weight coefficient α = 0.6, β = 0.3, γ = 0.1.

The constraint is that each note is struck by only one finger, and to avoid rapid fatigue for patients, the minimum time interval between two strikes of the same finger is ≥0.2 s. Using the Hungarian algorithm, the optimal matching solution is determined by finding an augmented path that minimizes the total cost with a time complexity of *O*(*n*^3^). For the application scenario in this study, the algorithm enables millisecond-level computation, ensuring real-time execution of finger assignment and seamless interaction between music-driven input and rehabilitation training.

#### 2.2.2. Mid-Level Coordination Layer: Motion Sequence Planning

To accurately position the hand rehabilitation robot on the target key, coordinated movement in both the lateral (X-axis) and longitudinal (Y-axis) directions is required. Based on the layout of a 61-key electronic keyboard, a two-stage path planning strategy is proposed, consisting of a coarse positioning stage and a fine adjustment stage.

In the first stage, the horizontal sliding platform rapidly moves to the region of the target key, with a positioning tolerance of ±5 mm. In the second stage, the vertical sliding platform finely adjusts its position to accommodate the spatial differences between black and white keys. The movement of the sliding platform in the XY plane is governed by the following differential equation:

(20) $$\left\{\begin{array}{l} \dot{x}=v_{x}\\ \dot{y}=v_{y}\\ {\dot{v}}_{x}=\frac{1}{\tau }(K_{u}u_{x}-v_{x})\\ {\dot{v}}_{y}=\frac{1}{\tau }(K_{u}u_{y}-v_{y}) \end{array}\right.$$

Among them, τ = 0.15 s is the motor response time constant, and $K_{u}$ = 0.8 m/(s·V) is the voltage speed gain coefficient. Once the sliding platform reaches the target position, the hand rehabilitation robot must actuate a specific finger to press the key. To ensure precise timing control, an event-triggered synchronization mechanism is implemented. The triggering conditions are set as positioning error ≤ 0.5 mm and velocity ≤ 1 mm/s. Upon satisfying these conditions, an additional 50 ms delay is introduced to eliminate mechanical vibrations caused by platform translation before initiating the finger key-pressing action.

#### 2.2.3. Low-Level Execution Layer: High-Precision Motion Control

To achieve high-precision positioning of both the horizontal (X-axis) and vertical (Y-axis) sliding platforms, a PID feedforward compensation control strategy is implemented [26]:

(21) $$u(t)=K_{p}e(t)+K_{i}\int_{0}^{t}e(\tau )d\tau +K_{d}\frac{de}{dt}+F_{\mathrm{feedforward}}$$

The feedforward term $F_{\mathrm{feedforward}}$ is calculated based on the motor dynamics model:

(22) $$F_{\mathrm{feedforward}}=J{\ddot{x}}_{d}+B{\dot{x}}_{d}$$

J = 0.2 kg·m^2^ is the equivalent moment of inertia, B = 0.5 N·s/m is the damping coefficient.

The key tapping requires simultaneous control of position (to ensure the depth of pressing) and force (to avoid overload). This study uses an impedance–admittance hybrid control strategy to perform force–position hybrid control on the key-tapping action of a hand rehabilitation robot. Dynamically adjust the desired position based on the key reaction force $F_{\mathrm{key}}$:

(23) $$x_{d}=x_{0}+\frac{F_{\mathrm{key}}}{K_{\mathrm{virtual}}}$$

$K_{\mathrm{virtual}}$ = 150 N/m is the virtual stiffness, simulating the force–position characteristics of real piano keys. Track and adjust the position through the PD controller:

(24) $$F_{\mathrm{cmd}}=K_{p}(x_{d}-x)+K_{d}({\dot{x}}_{d}-\dot{x})$$

$F_{\mathrm{cmd}}$ tracks the adjusted position through the PD controller, setting the parameter to $K_{p}$ = 120 N/m, $K_{d}$ = 15 N·s/m.

The position information is obtained in real time from the encoder of the pushrod motor, which provides a precision of ±0.01 mm. Traditionally, force information is acquired by installing force sensors at the fingertips to measure the key-pressing force. However, this approach increases wiring complexity and compromises the convenience and practicality of the system. To address these challenges, we propose a stroke feedback-based force estimation method, which calculates real-time resistance based on the velocity of the push rod motor before and after contacting the key and the duration of the impact.

Let *V*_0_ be the motor velocity before key contact; when the finger touches the piano key, the motor experiences a negative acceleration *a* due to resistance. Using the numerical differentiation method, the acceleration *a* can be computed as follows:

(25) $$a(t)\approx \frac{v(t)-v(t-\Delta t)}{\Delta t}=\frac{{\left.\frac{dx}{dt}-\frac{dx}{dt}\right|}_{t-\Delta t}}{\Delta t}$$

According to Newton’s second law, the real-time force can be obtained as follows:

(26) $$F=m_{\mathrm{eff}}a$$

F represents the contact force between the finger and the piano key. $m_{\mathrm{eff}}$ is the equivalent mass of the motor transmission system, which takes into account the transmission ratio of the push rod motor as well as the equivalent mass of the finger. To ensure the safety of patients with arthritis or limited finger movement, the system incorporates an impedance–admittance hybrid control strategy and real-time force feedback monitoring. The applied force of each actuator is continuously monitored, and excessive force triggers an automatic reduction in actuator output.

## 3. Experiment and Result Analysis

### 3.1. Comparative Experiment Setup

To verify the rehabilitation effect of this device in music-driven occupational therapy, this study designed a randomized controlled experiment, aiming to compare the differences in the rehabilitation effects between music therapy combined with occupational therapy (experimental group) and traditional repetitive passive training (control group) for patients with upper-limb dysfunction. The experimental protocol has been approved by the Ethics Committee of Chengde Medical University (Approval Number: 2025002), and informed consent was obtained from all subjects involved in the study. The experimental design fully considers the scientific nature and operability of rehabilitation training. Participants were screened through strict inclusion and exclusion criteria, and the stratified randomization method was used to ensure the balance of baseline characteristics between the experimental group and the control group.

#### 3.1.1. Experimental Design

**Figure 8:**
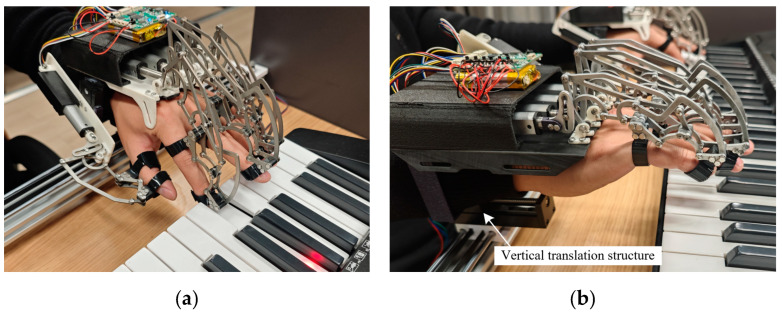
The specific settings for the hand rehabilitation training of the patients in the experimental group: (**a**) The hand state when striking the piano keys; (**b**) The hand state when switching between striking the white and black keys.

The experiment adopted a two-group parallel control design, divided into an experimental group and a control group, with five patients in each group. The experimental group used this device for music-driven occupational therapy training, and the experimental setup is shown in Figure 8. The patients in the experimental group must wear the hand rehabilitation robot on both hands, and their bilateral upper limbs are passively driven by the proposed device to perform piano playing. The control group only undergoes CPM for the affected upper limb.

The training content was piano playing, covering single notes, chords, and simple melodies. The control group, on the other hand, used the same device for repetitive passive training continuous passive motion (CPM). The training content was the movement of the shoulder and elbow joints and fingers along a fixed trajectory without musical elements. The training period was 8 weeks, five times a week, and 30 min each time. The training intensity was dynamically adjusted according to the patient’s baseline ability. The initial intensity was 50% of the maximum ability, increasing by 10% every week. The experimental group enabled the music-driven function of the device, including modules such as note analysis, fingering assignment, and dynamic parameter adjustment, while the control group turned off the music-driven function and only enabled the CPM mode to execute the preset fixed movement trajectory.

#### 3.1.2. Participant Recruitment and Grouping

The recruitment of participants strictly followed the inclusion and exclusion criteria. The inclusion criteria were as follows: aged between 18 and 65 years old; diagnosed with upper-limb dysfunction caused by stroke or spinal cord injury (Fugl–Meyer score ≤ 50); voluntarily participating and signing the informed consent form. The exclusion criteria included having severe cardiovascular diseases or contraindications to exercise, having a previous background in music training (which may affect the experimental results), and being unable to complete the 8-week training program. The stratified randomization method was adopted. According to the baseline Fugl–Meyer scores, the participants were divided into two groups: the mild group (20–35 points) and the moderate group (36–50 points). Within each group, participants were randomly assigned to either the experimental group or the control group. Finally, 10 patients were included, with five patients in the experimental group and five patients in the control group. There were no significant differences in the baseline characteristics between the two groups (*p* > 0.05, Table 1).

#### 3.1.3. Evaluation Indicators and Methods

The evaluation indicators of the experiment include primary indicators and secondary indicators. The primary indicators are the upper-limb motor function and the ability to perform daily living activities. The Fugl–Meyer assessment for the upper extremity (FMA-UE, with a range of 0–66) and the box and block test (BBT, with a range of 0–40) were used to evaluate the rehabilitation of the upper limb and hand motor functions [27,28,29], respectively. The secondary indicators include finger flexibility and the subjective experience of patients. The NHPT and the rehabilitation motivation scale (RMS, with a range of 0–60) are used for evaluation [30,31,32]. The evaluation time points include the baseline evaluation (T0, before the start of the training), the mid-term evaluation (T1, 4 weeks after the start of the training), and the end-point evaluation (T2, 8 weeks after the start of the training).

#### 3.1.4. Data Collection and Analysis

The collection of experimental data strictly follows a standardized process to ensure the reliability and comparability of the data. All clinical scale evaluations (FMA-UE, BBT, NHPT, RMS) are measured by independent rehabilitation therapists before, during, and after the subjects’ training to eliminate observer bias. The kinematic data are automatically recorded by the rehabilitation device, including the movement trajectories of the fingers, the stroke of the push rod motor, the speed, and the striking force estimated based on the speed change. During the training process, the movement trajectory of each key press is recorded at a sampling rate of 100 Hz to ensure sufficient spatio-temporal resolution. In addition, the device stores the patients’ training data, which is convenient for analyzing the changing trends of finger movement control.

The data analysis is carried out using SPSS 27.0 and MATLAB 2023B. All continuous variables are expressed as mean ± standard deviation, and the normality test is performed through the Shapiro–Wilk test. The main analysis methods include a two-way repeated measures analysis of variance (Group × Time) to evaluate the changing trends of motor function indicators over time. The paired *t*-test or the Wilcoxon signed-rank test is used to compare the within-group changes before and after the training, and the independent samples *t*-test or the Mann–Whitney U test is used to compare the differences between the experimental group and the control group at the end of the training. In addition, to explore the relationship between kinematic parameters (such as the speed of the push rod motor and the striking force) and functional evaluation indicators (such as FMA-UE, NHPT, BBT), Pearson correlation analysis is adopted, and a linear regression model is used to analyze their predictive effects on the rehabilitation process. The significance level of all statistical tests is set at *p* < 0.05, and the effect size (Cohen’s d) is calculated to quantify the magnitude of the intervention effect [33].

### 3.2. Analysis of Experimental Results

**Figure 9:**
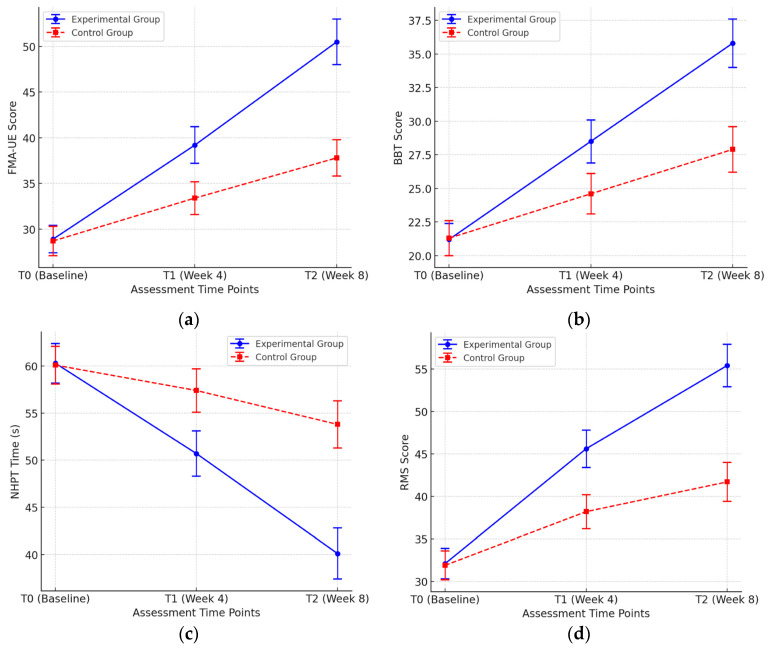
The rehabilitation evaluation data of the experimental group and the control group: (**a**) Comparison of upper-limb motor function; (**b**) Comparison of finger flexibility; (**c**) Comparison of fine finger movement control ability; (**d**) Comparison of patients’ subjective experience.

To evaluate the differences in the rehabilitation effects of music-driven occupational therapy and traditional repetitive passive training on patients with upper-limb dysfunction, this study systematically analyzed the evaluation data of the experimental group and the control group at three time points: baseline (T0), mid-term (T1), and end-point (T2). By comparing the changes in the indicators such as upper-limb motor function, ability to perform daily living activities, finger flexibility, and the subjective experience of patients between the two groups, as shown in Figure 9, the significant advantages of music-driven occupational therapy in terms of rehabilitation effects were verified.

In terms of upper-limb motor function (FMA-UE score), the patients in the experimental group significantly improved from T0 (28.9 ± 2.3) to T2 (50.5 ± 2.5), while the FMA-UE score of the control group only increased from 28.7 ± 3.1 to 37.8 ± 2.9. The two-way repeated measures analysis of variance (ANOVA) showed that the improvement range of the experimental group was significantly higher than that of the control group (*p* < 0.001), indicating that the music-driven training could effectively promote the recovery of upper-limb motor ability. In terms of hand flexibility (BBT score), the patients in the experimental group had an average increase of 14.6 points after 8 weeks of training (T2), while the increase in the control group was only 6.6 points (*p* = 0.002). This result shows that occupational therapy combining music rhythm and multi-joint coordinated training can enhance the hand coordination of patients more effectively than simple repetitive passive training. In terms of fine finger movement control ability (NHPT test), the time required for patients in the experimental group to complete the task decreased from T0 (60.3 ± 3.2 s) to T2 (40.1 ± 2.8 s), while the control group only decreased from 60.1 ± 2.9 s to 53.8 ± 3.1 s during the same period. The statistical analysis shows that the improvement in finger flexibility of the experimental group was significantly better than that of the control group (*p* = 0.004), proving the positive impact of music-assisted training on the coordinated movement ability of fingers. In addition, in terms of the subjective rehabilitation experience of patients (RMS score), the rehabilitation motivation score of patients in the experimental group increased from T0 (32.1 ± 3.4) to T2 (55.4 ± 3.8), while the control group only increased from 31.9 ± 3.2 to 41.7 ± 4.1. The statistical analysis (*p* < 0.001) shows that music-driven training can significantly increase training interest and long-term rehabilitation compliance of patients.

## 4. Discussion

This study systematically evaluated the effects of music-driven occupational therapy compared to traditional repetitive passive training on upper-limb rehabilitation in patients with motor dysfunction. The results demonstrated that the experimental group, which received music-driven therapy, exhibited significantly greater improvements in motor function, dexterity, fine motor control, and rehabilitation motivation compared to the control group undergoing CPM training. These findings suggest that integrating musical elements into rehabilitation not only enhances motor recovery but also increases patient engagement and adherence to therapy.

### 4.1. Upper-Limb Motor Function Improvement

The FMA-UE scores showed a significant increase in the experimental group compared to the control group, confirming that music-driven occupational therapy promotes superior recovery of upper-limb function. The observed improvement in the experimental group (from 28.9 ± 2.3 at T0 to 50.5 ± 2.5 at T2) suggests that the combination of music rhythm, active engagement, and multi-joint coordination training accelerates neuromotor adaptation. The control group, which only received repetitive passive motion training, also exhibited improvements but at a much slower rate (28.7 ± 3.1 at T0 to 37.8 ± 2.9 at T2). These results align with previous studies indicating that active movement therapy induces greater neuroplasticity than passive motion alone. The presence of rhythm and structured movement sequences in piano playing may facilitate sensorimotor integration, further enhancing motor recovery.

### 4.2. Manual Dexterity and Fine Motor Control

The BBT and NHPT results indicate that the experimental group exhibited significantly better improvements in both gross and fine manual dexterity. The BBT scores increased by 14.6 points in the experimental group vs. only 6.6 points in the control group, while NHPT completion time decreased by 20.2 s in the experimental group vs. only 6.3 s in the control group. These findings highlight the advantage of music-driven movement training, which requires patients to perform controlled, rhythmic, and goal-directed hand movements. Unlike passive movement training, which lacks cognitive engagement, music-assisted therapy demands continuous visual–motor coordination, promoting motor learning and increasing patient motivation.

### 4.3. Patient Motivation and Rehabilitation Adherence

One of the most striking findings was the improvement in rehabilitation motivation, as reflected in the RMS scores. The experimental group showed a substantial increase in RMS scores from 32.1 ± 3.4 at T0 to 55.4 ± 3.8 at T2, whereas the control group only increased from 31.9 ± 3.2 to 41.7 ± 4.1. The statistically significant difference (*p* < 0.001) between groups supports the hypothesis that incorporating music and interactive tasks into rehabilitation enhances intrinsic motivation and therapy adherence. This aligns with the previous literature indicating that emotionally engaging tasks improve neurorehabilitation outcomes by fostering long-term participation and compliance.

### 4.4. Clinical Implications and Future Directions

The findings of this study provide strong evidence supporting the integration of music-driven occupational therapy in upper-limb rehabilitation programs. The structured yet adaptable nature of piano-based movement training ensures both progressive motor recovery and sustained patient interest. However, future research should focus on larger sample sizes and extended follow-up periods to assess the long-term effects of music-assisted rehabilitation. Additionally, incorporating more complex musical elements or adaptive difficulty levels could further optimize motor learning and therapeutic outcomes.

## 5. Conclusions

This study designed and validated a novel upper-limb rehabilitation device that integrates piano playing with rehabilitation training, proposing an innovative music-driven occupational therapy model. Experimental results demonstrate that the device significantly outperforms traditional repetitive passive training devices in improving upper-limb motor function, daily living ability, finger dexterity, and patients’ subjective experience. The proposed mechanical structure design addresses the challenge of adapting to the spatial distribution of piano black and white keys through a multi-degree-of-freedom coordinated motion scheme. The hierarchical control strategy, incorporating music-to-mechanical action mapping and dynamic parameter adaptation, achieves personalized and intelligent training processes. A randomized controlled trial (experimental group vs. control group, *n* = 5/group) confirmed the clinical effectiveness of the device. The experimental group showed a 74.7% improvement in Fugl–Meyer Assessment scores (50.5 ± 2.5), significantly higher than the control group’s 31.7% (37.8 ± 2.9). The experimental group’s BBT scores increased by 14.6 points, markedly surpassing the control group’s 6.6-point improvement. The nine-hole peg test completion time decreased by 20.2 s in the experimental group, compared to only 6.3 s in the control group. The experimental group’s RMS scores improved by 72.6% (55.4 ± 3.8), indicating that music-driven tasks significantly enhance patient engagement. These results confirm that the proposed upper-limb rehabilitation training device, which combines piano playing with occupational therapy, effectively promotes neuroplasticity reconstruction and enhances the efficiency, enjoyment, and immersion of rehabilitation training.

## Figures and Tables

**Table 1:** Baseline Characteristics Table.

Characteristic	Experimental Group (*n* = 5)	Control Group (*n* = 5)	*p*-Value
Age (years)	59.4 ± 2.2	56.2 ± 4.8	0.27
Sex (M/F)	3/2	2/3	0.63
Course of disease (months)	6.1 ± 2.2	5.9 ± 2.1	0.58
FMA-UE (score)	28.9 ± 2.3	28.7 ± 3.1	0.92
BBT (score)	21.2 ± 3.7	21.3 ± 3.3	0.98
Characteristic	Experimental Group (*n* = 5)	Control Group (*n* = 5)	*p*-value

## Data Availability

Details of data availability are available from the first author on request.
